# Scaffold‐Hunting From Pyriproxyfen: A Biochemoinformatic Route to Novel Insecticidal Leads

**DOI:** 10.1155/bmri/6490830

**Published:** 2026-10-21

**Authors:** Edla L. V. S. dos Santos, Ryan da S. Ramos, Rai C. Silva, Luciane B. da Silva, Lúcio R. Lima, Ruan S. Bastos, Aderaldo V. da Silva, Sylviah S. Mwanza, Njogu M. Kimani, José M. Espejo-Román, Joaquín M. C. Rosa, Raimundo N. P. Souto, Cleydson. B. R. Santos

**Affiliations:** ^1^ Postgraduate Program in Pharmaceutical Innovation Postgraduate Department Federal University of Amapá Macapá Brazil unifap.br; ^2^ Laboratory of Modeling and Computational Chemistry Department of Biological and Health Sciences Federal University of Amapá Macapá Brazil unifap.br; ^3^ Laboratory of Arthropoda Department of Biological and Health Sciences Federal University of Amapá Macapá Brazil unifap.br; ^4^ Postgraduate Program in Biotechnology and Biodiversity-Network BIONORTE Postgraduate Department Federal Univesity of Amapá Macapá Brazil; ^5^ Postgraduate Program in Medicinal Chemistry and Molecular Modeling Departments of Health Sciences Exact and Natural Sciences Biological and Technological Sciences Federal University of Pará Belém Brazil ufpa.br; ^6^ International Center of Insect Physiology and Ecology (icipe) Nairobi Kenya; ^7^ Department of Physical Sciences University of Embu Embu Kenya embuni.ac.ke; ^8^ Faculty of Pharmacy Department of Pharmaceutical and Organic Chemistry University of Granada Campus of Cartuja Granada Spain ugr.es

**Keywords:** acetylcholinesterase, ADME/tox, *Aedes aegypti*, insecticide resistance, juvenile hormone receptor, virtual screening

## Abstract

Vector‐borne diseases remain a critical global health concern, particularly in tropical and subtropical regions, where socioeconomic conditions often favor vector proliferation. Pyriproxyfen, a juvenile hormone analog used as a larvicide against *Aedes aegypti*, is known for its fast‐acting efficacy, low toxicity, and favorable environmental profile. Because of these properties, pyriproxyfen was chosen as a reference scaffold to explore structurally and electrostatically similar compounds through ligand‐based virtual screening. The ChemBridge DIVERSet‐CL database (≈ 100,000 compounds) was used, applying ROCS for shape similarity and EON for electrostatic potential comparison. A total of 35 top‐ranked molecules were identified based on superior pharmacokinetic and toxicological properties compared with the reference. Subsequently, molecular docking analyses were performed to assess binding affinity and interaction modes with selected biological targets: acetylcholinesterase (AChE) and juvenile hormone‐binding proteins from various organisms. Two compounds, LMQC3 and LMQC23, demonstrated strong binding affinities to AChE enzymes from *Drosophila melanogaster* (−11.4 and −10.6 kcal/mol), *Anopheles gambiae* (−10.19 and −10.21 kcal/mol), and humans (−9.91 and −9.95 kcal/mol). Additionally, they exhibited high affinity toward the juvenile hormone protein in *A. aegypti* (−11.83 and −11.94 kcal/mol), surpassing reference ligands. Both molecules established key interactions with catalytic residues, supporting their potential dual inhibitory activity. These findings indicate that LMQC3 and LMQC23 are potential promising insecticidal candidates with multitarget potential, showing favorable predicted target‐binding profiles and encouraging in silico pharmacokinetic and toxicity characteristics. Their development could contribute to next‐generation mosquito control strategies and help mitigate the spread of arbovirus‐related diseases.

## 1. Introduction

Arthropods, especially some insects of the family Culicidae, can transmit serious diseases caused by viruses, bacteria and protozoa, significantly impacting health systems. The genera with the largest number of known vectors of the Culicidae family are: *Aedes*, *Anopheles*, and *Culex*, with emphasis on the species *Aedes aegypti*, the primary vector for illnesses including dengue, yellow fever, Zika, and chikungunya [1]. In addition to these, other diseases are transmitted by different species and can affect humans, such as malaria, Oropouche, encephalitis, filariasis, and West Nile fever [2, 3]. Most of these vectors are concentrated in tropical countries, where environmental and social conditions are favorable to their proliferation. Given the absence of vaccines for some of these diseases, the adoption of prophylactic measures becomes essential for vector control. Some vector control strategies include educational programs, behavior change, and the use of insecticides. However, overreliance on synthetic chemical insecticides has fostered the development of resistant populations, ultimately increasing the frequency of these disease cases [4–8].

As there are still no vaccines or specific medications to prevent some of the diseases transmitted by mosquitoes, the development of insecticides that are selective and specific to these vectors is essential. Despite this, the vast majority of insecticides currently on the market present some degree of toxicity to the environment and other living organisms. Therefore, it is essential to develop compounds that are more selective and specific for target insects, while being safe for hosts and the ecosystem [7, 9, 10]. In view of this, alternative control approaches have been explored, including physical, biological, genetic, and chemical methods [11, 12]. In this context, studies in medicinal chemistry and molecular modeling have played a fundamental role in identifying promising molecules for the development of new insecticides. These compounds act in a targeted manner on specific insect target receptors, making interventions more selective and effective [6, 9, 10, 13].

The use of insect growth regulators (IGRs) for mosquito control has been receiving much attention as they disrupt the critical developmental stages of the insect. Moreover, they usually have lower mammalian toxicity than neurotoxic insecticides. These compounds are derived from biological sources such as viruses, bacteria, and fungi offering a more sustainable path for vector management [14]. An example of an IRG is pyriproxyfen (IUPAC: 2‐[1‐methyl‐2‐(4‐phenoxyphenoxy)ethoxy]pyridine; C_20_H_19_NO_3_). It is widely used as a larvicide against the *A. aegypti* mosquito, and has been endorsed by the World Health Organization (WHO) for use at low concentrations in drinking water since the 1970s. It features a central phenoxyphenoxy core linked to a pyridine ring via a 1‐methyl‐2‐ethoxy bridge. It shows high chemical stability across a wide pH range (pH 4, 7, and 9) and low volatility (see Table S1). In soil and water, it undergoes rapid photolytic degradation. It is a colorless‐to‐white crystalline solid with low water solubility and a strong propensity to adsorb onto soil organic matter and aquatic sediments. It exhibits low mobility and low potential for groundwater contamination. It is known for its rapid larvicidal action and environmental residual action, although its toxicity is relatively low when compared with other compounds of the same class [13, 15–18]. Pyriproxyfen, a pyridine‐based compound, emerged in the 1990s as a commercial insecticidal solution from Sumitomo Chemical Co. Ltd. This aromatic, nonterpenoidal compound is derived from phenyl carbonyl, in which the aliphatic chain has been replaced by a pyridyl oxyethylene (Figure 1). This compound functions as a potent juvenile hormone (JH) mimic, disrupting the hormonal equilibrium of insects. By arresting embryonic development and preventing the transition to adulthood, it inhibits the formation of essential mature features such as wings and reproductive organs [19]. Consequently, the insect remains trapped in an immature larval or pupal state, and any individuals that reach maturity are rendered sterile [20].

**Figure 1:**
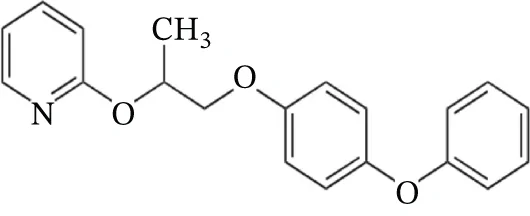
2D molecular structure of pyriproxyfen molecule.

Recent advances in computational chemistry and chembioinformatics have accelerated insecticide discovery. Ligand‐based virtual screening is used to identify molecules with physicochemical, three‐dimensional shape, and electrostatic properties similar to established insecticidal scaffolds. Molecular docking is subsequently used to provide insights into their potential interactions with biologically relevant targets. In silico ADME and toxicity prediction are then used to filter compounds with more favorable pharmacokinetic and safety profiles. This reduces the number of candidates requiring experimental evaluation. Past studies have demonstrated the utilization of these tools and workflows in identifying potential insecticidal compounds. However, these methods are frequently applied independently or focused on a single molecular target or activity endpoint. In this study, we integrated a computational strategy involving shape and electrostatic similarity in addition to pharmacokinetic and toxicity profiling, biological‐activity prediction, and multitarget molecular docking.

This study is a continuation of our exploration of novel pesticides [21], to identify novel insecticidal candidates inspired by the chemical features of pyriproxyfen (Figure 2). The ChemBridge DIVERSet‐CL library was screened using molecular shape and electrostatic similarity. The resultant hits were subjected to pharmacokinetic and toxicity profiling, biological‐activity prediction, and molecular docking against acetylcholinesterase (AChE) and JH‐related proteins. The study further compared the predicted binding of prioritized candidates with mosquito and human AChE. In the end, we identified LMQC3 and LMQC23 as promising potential candidates that can guide subsequent experimental validation to establish the predicted insecticidal efficacy.

**Figure 2:**
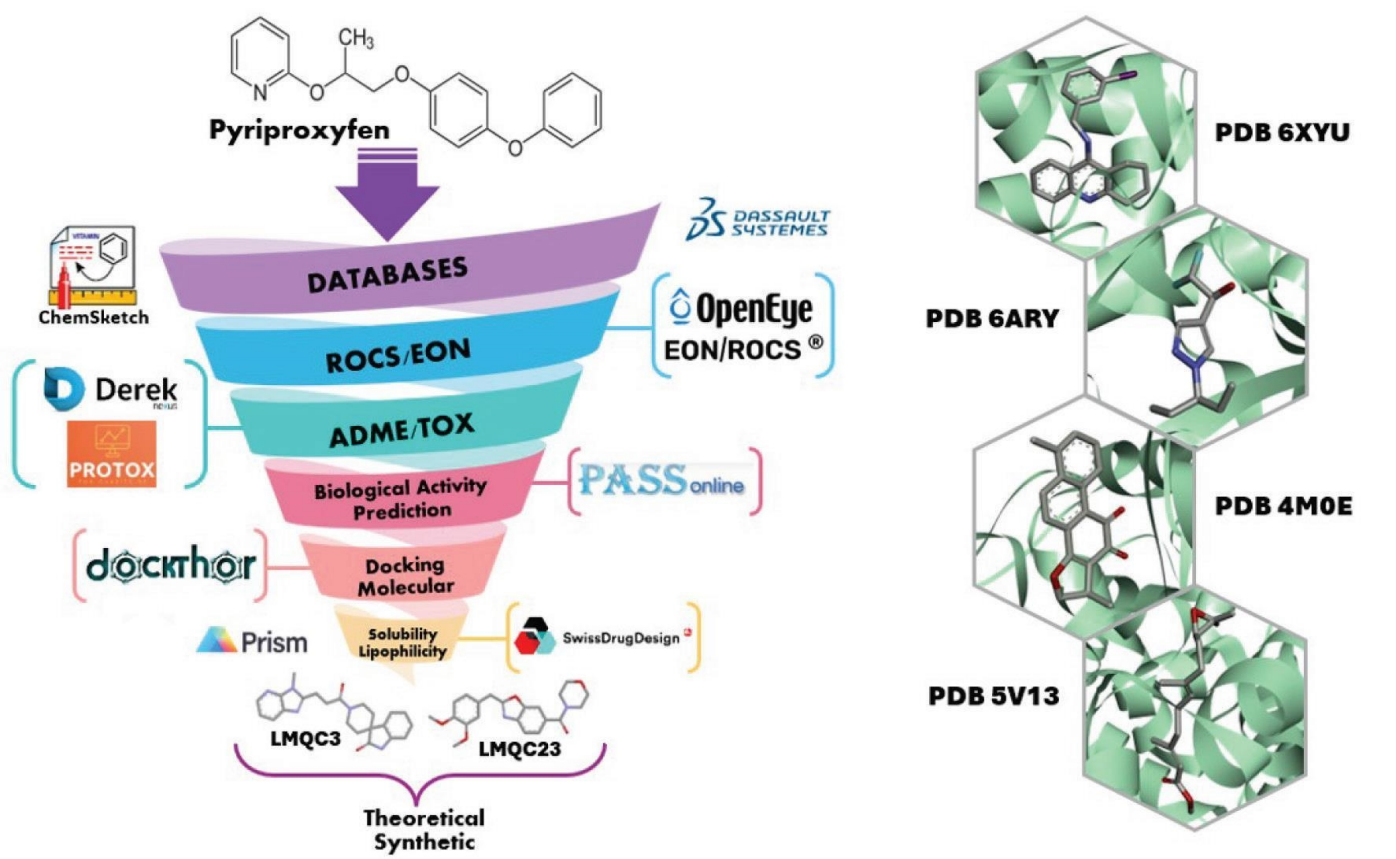
Virtual screening workflow, showing the main methodological steps, computational tools and PDBs used in the study.

## 2. Materials and Methods

### 2.1. Pivot Compound

Since the bioactive conformation of pyriproxyfen has not yet been structurally determined, its most rigid conformation based on the available crystallographic structure according to Kang et al. [22], was used as input file for the virtual screening analysis by structural and electrostatic similarity, with the Rapid overlay of chemical structures (ROCS) and Electrostatic Similarity (EON) software [23–25].

### 2.2. Generation of a Library of Conformers

In this study, the virtual screening stage was performed on the ChemBridge database (DIVERSet‐CL subcollection) (https://chembridge.com/). This database contains approximately 100,000 freely accessible compounds in its diversity library, mainly used in scientific research, and is updated regularly. Three hundred conformers for each molecule in the database were generated using the MMFF94 force field on OMEGA software (OpenEye Scientific Software, Santa Fe, New Mexico) (http://www.eyesopen.com). The conformer generation process was constrained by a maximum deformation energy of 9 kcal/mol relative to the global minimum, with a structural diversity threshold defined by a root mean square deviation (RMSD) of 0.6 Å. [24, 25]

### 2.3. Virtual Screening

#### 2.3.1. Rapid Superposition of Chemical Structures (ROCS)

Virtual screening was performed on the ChemBridge DIVERSet‐CL library by employing Gaussian‐based shape approximations. This method determined the optimal alignment of candidate molecules by calculating their maximum overlapping volumes relative to the reference compound [25]. Using the ROCS software′s Gaussian‐based algorithm, we performed a three‐dimensional overlap analysis between the database entries and the pyriproxyfen reference structure. This screening process allowed for the ranking of candidates based on structural similarity, from which the top 2000 lead structures were selected for further evaluation [24–26].

#### 2.3.2. Electrostatic Similarity (EON)

Electrostatic Tanimoto indices were calculated using EON software (https://www.eyesopen.com/eon), with partial charges assigned via MMFF94 energy minimization to compare the electronic similarity of the database hits to pyriproxyfen. [24–26]. Candidates were ranked based on Tanimoto electrostatic scores, which scale from 1 (representing identical electronic profiles) to negative values for mismatched charges. Using a single, rigid, lowest‐energy conformer of pyriproxyfen, derived from its crystallographic structure, as the reference, we evaluated the electrostatic overlap of the database. Results were sorted by the “ET_combo” metric, which integrates shape and electronic similarity, ultimately narrowing the selection to the 100 highest‐ranked molecules.

### 2.4. In Silico Pharmacokinetic and Toxicological Properties

The compounds selected previously were analyzed by QikProp software to determine their pharmacokinetic properties [27]. The generated parameters allowed choosing the candidate with 95% similarity to drugs described in the literature, ensuring data reliability [28]. The toxicological profiles of the candidate molecules were assessed via DEREK, an in‐silico platform specialized in predicting toxicity during the drug design process [29–33].

Biological activities were predicted using the PASS web server (http://www.pharmaexpert.ru/passonline) [34]. Here, we identified the potential biological effects of compounds derived strictly from their molecular formulas. The analysis process employed Multilevel Neighboring of Atoms (MNA) descriptors, operating on the principle that a molecule′s specific chemical architecture is the primary determinant of its biological activity [33, 35–37]. Only compounds with indications of insecticidal and anticholinesterase activities were selected for the subsequent analysis.

### 2.5. Prediction of Toxicity Risk

ProTox (http://tox.charite.de/protox_II/), a tool valuable for identifying undesirable toxic properties present in the molecules tested, was employed here [38, 39]. Predictions were generated by comparing the functional groups of the candidate molecules against a comprehensive database of established in vitro and in vivo data [21]. Toxicological parameters were established, including the determination of *L* *D*_50_ values (expressed in mg/kg) and the subsequent assignment of toxicity classifications.

### 2.6. Molecular Docking

Following the initial screening, only top‐ranked candidates demonstrating favorable pharmacokinetic, toxicological, and biological activity profiles were progressed to molecular docking. These simulations evaluated the binding efficacy of the ligands through free energy (*Δ* *G*) calculations, accompanied by a detailed analysis of their binding modes, spatial orientations, and overall affinity for the chosen biological targets.

#### 2.6.1. Selection of Enzyme and Inhibitor Structures

Insecticidal agents target various biological pathways, with two primary mechanisms involving the inhibition of the enzyme AChE and the disruption of JH signaling [40–42]. AChE remains a primary molecular target for various pesticides, specifically organophosphates and carbamates. Although these chemical classes possess significant toxicity profiles, they continue to see extensive application in both large‐scale agriculture and residential pest control [41–43]. The selection of AChE as a target is supported by the availability of high‐resolution crystallographic structures, both in its apo‐form and when bound to ligands. Concurrently, Juvenile Hormone III (JHIII) serves as a fundamental regulator of insect ontogeny and fecundity. In mosquitoes, JHIII is indispensable for oocyte development and male reproductive patterns, though the mechanisms governing its systemic distribution and postsecretion regulation are not yet fully understood. [40].

A comparative molecular modeling analysis was conducted using the human AChE crystal structure (PDB ID: 1YL) complexed with Dihydrotanshinone I. This study assessed binding affinities, free energy values, and specific interactions with amino acid residues. The use of the human enzyme is supported by literature indicating high sequence similarity between human and insect AChE variants [42, 43]. The lack of an elucidated crystallographic structure for *A. aegypti* AChE inspired the selection of these targets.

The crystallographic structure of *Drosophila melanogaster* AChE bound to the tacrine‐derived inhibitor (I40), was retrieved from the Protein Data Bank (PDB ID: 6XYU). This structure provides a structural template with a resolution of 2.5 Å [42]. The crystal structure of recombinant human hAChE in complex with Dihydrotanshinone I was retrieved from the Protein Data Bank (PDB ID: 4M0E). This model, resolved at 2.0 Å, provides a high‐fidelity template for analyzing ligand‐receptor interactions [26, 44]. The crystal structure of *Anopheles gambiae* AgAChE, in complex with the ligand BT7, was retrieved from the Protein Data Bank (PDB ID: 6ARY). This structure, resolved at 2.2 Å, serves as a species‐specific template for evaluating insecticidal interactions [45]. The high‐resolution (1.8 Å) crystallographic structure of the JH complexed with methyl (2E,6E)‐9‐[(2R)‐3,3‐dimethyloxiran‐2‐yl]‐3,7‐dimethylnon‐2,6‐dienoate (JHIII) was retrieved from the Protein Data Bank (PDB ID: 5 V13) [26, 46]. Compounds I40, 1YL, BT7, JHIII, and pyriproxyfen served as reference inhibitors for the molecular docking simulations. These studies were conducted in accordance with a robust and validated protocol previously established in our research group [47–51]. The molecular structures of the molecules in complex with AChE and JH are shown in Figure 3.

**Figure 3:**
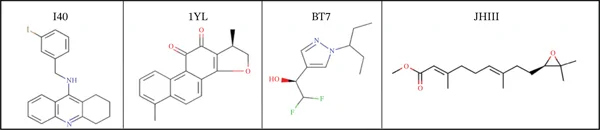
Structures of AChE and juvenile hormone inhibitors: 9‐(3‐iodobenzylamin)‐1,2,3,4‐tetrahydroacridine (140), Dihydrotanshinone I (1YL), (1S)‐2,2‐difluoro‐1‐[1‐(pentan‐3‐yl)‐1H‐pyrazol‐4‐yl]ethan‐1‐ol (BT7), and methyl(2E,6E)‐9‐[(2R)‐3,3‐dimethyloxyxiran‐2‐yl]‐3,7‐dimethylnone‐2,6‐dienoate (JHIII), used as control in this study.

#### 2.6.2. Molecular Docking

The protein structure and the ligands were prepared with Discovery Studio 5.0 software [52]. AChE (*D. melanogaster*, *A. gambiae*, and *H. sapiens*) and JH (*A. aegypti*) were used in complex with specific ligands in DockThor. The molecular docking protocol validation was performed by comparing the crystallographic ligand with the best redocked conformation (PDB ID: 6XYU, 6ARY, 4M0E and 5 V13).

The *x*, *y*, and *z* coordinates of the targets were determined by averaging within the active site region. The grid′s central coordinates are shown in Table 1. The energy function score was used to assess the binding free energy (*Δ* *G*) between ligands and receptor amino acid residues. Conformational analysis was also performed to select the optimal binding free energy using DockThor. Visualizations and measurements of distances in inhibitor‐enzyme interactions were conducted with Discovery Studio 5.0.

**Table 1:** Molecular docking target protein data.

Enzyme	Inhibitor	Grid coordinates	Grid points (size)
AChE (PDB 6XYU)	9‐(3‐Iodobenzylamino)‐1,2,3,4 tetrahydroacridine	*x* = 33.13	20
		*y* = 67.65	20
		*z* = 9.89	20
AChE (PDB 4M0E)	Dihydrotanshinone I	*x* = −17.2	20
		*y* = −42.38	20
		*z* = 25.59	20
AChE (PDB 6ARY)	Difluoromethyl ketone inhibitor: (1S)‐2,2‐difluoro‐1‐[1‐(pentan‐3‐yl)‐1H pyrazol‐4‐ylJethan‐1‐ol	*x* = −85.82	20
		*y* = 54.96	20
		*z* = −15.1	20

### 2.7. Lipophilicity and Water Solubility Prediction

This analysis aimed to obtain compound solubility data necessary for future in vivo assays, highlighting the importance of dilutions, solution preparation and types of chemical solvents used. The partition coefficient between n‐octanol and water (LogPo/w) is the main descriptor of lipophilicity. Consequently, employing a broader range of diverse predictive methodologies enhances the reliability and accuracy of the resulting consensus value of LogPo/w [21, 53–55].

The SwissADME web server (http://www.swissadme.ch/) offers a suite of five predictive methodologies designed to generate reliable data on potential drug candidates for subsequent biological evaluation. Among these, XLOGP3 serves as an atom‐based predictive model; it calculates lipophilicity by applying corrective factors to the LogP values of established reference compounds [56]. WLOGP represents an alternative atomistic approach that lacks corrective factors, instead adhering strictly to the fragmentation methodology introduced by Wildman and Crippen [57]. MLOGP is a topological estimation method that establishes a relationship between molecular structure and LogP across a diverse chemical space, including both pharmaceuticals and agrochemicals. This approach utilizes multiple linear regression analysis across 13 specific molecular descriptors to determine lipophilicity [58]. SILICOS‐IT is a hybrid method that uses 27 fragments and 7 topological descriptors calculated using FILTER‐IT software (Silicos Co., Antwerp, Belgium) (http://www.silicos-it.be/software). iLOGP represents a modern physics‐based approach that determines lipophilicity by calculating the free energy of solvation in both n‐octanol and water. This method utilizes the generalized Born implicit solvent model combined with solvent accessible surface area (GB/SA) calculations to derive its predictions [21, 59].

A molecule′s aqueous solubility (LogS) is a critical parameter for establishing the necessary dilution protocols and ensuring the appropriate administration of a compound during biological assays [60]. The SwissADME platform incorporates three distinct topological approaches for calculating aqueous solubility, including the ESOL (estimated solubility) [61], the Ali [62] and the SILICOS‐IT models (http://www.silicos-it.be/software.html). To make comparisons and arrive at a more assertive synthetic accessibility (SA) value for the compounds studied, the Ambit‐AS software was also used [63]. This software employs an additive scoring scheme based on four weighted molecular descriptors that account for distinct structural and topological characteristics. The algorithm evaluates a target compound by analyzing its molecular and stereochemical complexity, alongside the presence of fused or bridged ring systems. The resulting SA score is mapped to a scale of 0 to 100; a score of 100 represents maximum SA (indicating a straightforward synthesis), whereas a score of 0 signifies minimal accessibility, representing a molecule that is extremely difficult to synthesize [21, 33].

### 2.8. Theoretical Synthetic

The synthetic routes for compounds LMQC3 and LMQC23 were designed and adapted from methodologies described in patent literature, encompassing all reaction steps, required reagents, and experimental conditions for obtaining the final products.

## 3. Results and Discussion

In this study, we integrated a ligand‐ and structure‐based in silico workflow to identify potential insecticidal compounds with chemical features similar to pyriproxyfen. We sequentially applied ROCS shape similarity, EON electrostatic similarity, ADME and toxicity prediction, PASS bioactivity prediction, and molecular docking. From a library of approximately 100,000 compounds, we identified two candidates, LMQC3 and LMQC23. This workflow is consistent with previous studies that used pyriproxyfen as a reference structure for identifying new compounds with potential insecticidal activity. For instance, in an earlier study, pyriproxyfen‐based virtual screening of the ZINC database, identified ZINC00001624 as a potential inhibitor with predicted binding affinities of −10.5 kcal/mol and −10.3 kcal/mol against insect and human AChE, respectively. Additionally, ZINC00001021 was predicted to have a strong interaction with JH‐related targets [39]. Another pyriproxyfen‐based virtual screening of the Maybridge database identified potential and JH inhibitors that could be potential insecticides [64] . These past studies support the general suitability of pyriproxyfen as a reference starting point for insecticide discovery.

### 3.1. ROCS and Electrostatic Similarity (EON)

The cornerstone of ligand‐based drug design is the identification of chemically analogous compounds. This strategy aims to retrieve molecules from expansive databases that mirror both the structure and biological profile of a reference ligand. This approach is rooted in the similarity principle, which asserts that structurally related molecules are predisposed to demonstrating comparable biological activities [21, 65].

Ligand‐based virtual screening was performed using the OpenEye ROCS software. This platform facilitates the comparison of multiconformational libraries against a reference structure′s volume. The algorithm utilizes Gaussian functions centered on atoms to determine the optimal spatial overlap, evaluating both steric volume and chemical features to produce a unified alignment metric known as the Comboscore [66].

Using pyriproxyfen as a reference template, we conducted a shape‐based screening of the ChemBridge DIVERSet‐CL subcollection using the ROCS program. We applied the “Top2000/base” selection criterion to identify the 2000 molecules with the highest structural similarity to the model. These resulting structures were then exported as input files for subsequent electrostatic analysis in the EON software [67]. Following the initial alignment in ROCS (v3.4.2), we evaluated the electrostatic similarity of the candidate structures using EON (v2.3.2). By utilizing pyriproxyfen as the common reference, the program compared the electrostatic potential maps of the pre‐aligned molecules. This process identified compounds with analogous morphologies and electronic distributions based on Tanimoto index measurements. [21, 68]. Following the computational analysis, the screening process concluded with the isolation of the “Top100/base” candidates. These 100 structures represented the highest‐scoring molecules based on their structural and electrostatic similarity to the reference.

### 3.2. Toxicological and Pharmacokinetic Properties

Evaluating toxicological and pharmacokinetic properties is essential in the development of effective and safe insecticides. These analyses allow us to understand how compounds are absorbed, distributed, metabolized, and excreted in target organisms, in addition to predicting possible unfavorable effects on nontarget organisms and the environment. This approach contributes to the creation of more selective products, reducing toxicological risks and environmental impacts [69].

The pharmacokinetic properties are presented in Table 2 for 35 compounds selected through virtual screening, using the ROCS, followed by the calculation of electrostatic similarity with the EON software. The “stars” parameter reflects the number of property or descriptor values that do not fall within the 95% similarity range in relation to already established drugs. Higher star values indicate that a compound is less similar to an existing drug, compared with those with lower values [70]. For this parameter, molecules whose values are closest to 0 were selected.

**Table 2:** Pharmacokinetic properties of selected molecules.

Molecules	Star^a^	CNS^b^	MW^c^	Clog _P/w_^d^	Log_B/B_^e^	HBD^f^	HBA^g^	R5^h^	R3^i^
Normal range	0–5	−2 to + 2	< 500	−2.0 to 6.5	−3.0 to 1.2	< 5	< 10	Max. 4	Max. 3
Pyriproxyfen	1	1	321.375	5.616	0.052	0	2.2	1	0
LMQC200	0	−1	328.388	2.149	−0.912	1	7.0	0	0
LMQC145	0	1	325.428	3.584	0.019	1	5.0	0	1
LMQC74‐39	1	0	323.354	3.443	−0.262	1	5.5	0	0
LMQC60	1	0	347.376	3.894	−0.540	1	6.0	0	0
LMQC53	0	−1	389.456	2.515	−0.776	1	8.0	0	0
LMQC284	1	0	367.422	5.520	−0.457	1	3.2	1	1
LMQC38	0	−1	301.32	2.647	−0.764	1	5.0	0	0
LMQC281	0	−1	364.446	3.027	−0.701	2	6.2	0	0
LMQC3	0	−1	360.418	3.915	−0.939	1	6.0	0	1
LMQC112	0	−1	397.491	2.964	−0.944	2	6.7	0	1
LMQC261	0	−1	326.394	4.037	−0.786	0	3.7	0	0
LMQC215	1	0	377.463	4.859	−0.515	1	5.5	0	1
LMQC209	1	−1	357.414	4.347	−0.775	1	5.5	0	1
LMQC229	0	−1	387.453	2.246	−0.805	1	8.2	0	1
LMQC244	1	0	343.387	3.950	−0.451	1	5.5	0	0
LMQC279	0	−1	342.415	2.461	−0.855	1	7.0	0	0
LMQC264	1	−1	348.465	3.447	−0.854	1	6.5	0	0
LMQC122	0	−2	370.407	2.460	−1.427	0	8.2	0	0
LMQC104	0	−1	368.478	3.129	−0.822	2	5.7	0	1
LMQC255	1	−1	359.46	4.269	−0.947	2	4.0	0	1
LMQC97	0	−2	350.453	2.598	−1.094	2	5.2	0	0
LMQC8	1	0	332.445	1.689	−0.348	0	7.0	0	0
LMQC271	0	−1	374.441	3.819	−0.883	2	5.5	0	0
LMQC296	0	−1	378.43	2.133	−0.664	0	8.2	0	0
LMQC251	1	0	383.507	5.028	−0.611	1	5.5	1	2
LMQC19	0	−2	366.419	1.641	−1.047	1	8.0	0	0
LMQC23	0	−1	382.415	2.927	−0.261	0	8.2	0	0
LMQC196	0	−1	379.458	3.639	−0.698	0	7.7	0	0
LMQC208	0	−1	373.469	2.130	−0.367	0	8.0	0	0
LMQC20	0	−2	353.42	1.961	−1.164	2	7.7	0	1
LMQC79‐39	2	0	426.555	4.524	−0.654	1	6.7	0	1
LMQC115	0	−1	323.427	3.692	−0.922	2	4.0	0	0
LMQC250	1	0	389.474	4.440	−0.541	1	6.5	0	1
LMQC184	0	−1	353.423	1.620	−0.804	0	8.5	0	0
LMQC46	0	−1	353.423	2.650	−0.733	0	7.0	0	0

^a^Number of computed properties which fall outside the required range for 95% of known drug.

^b^Activity in the central nervous system.

^c^Molar weight.

^d^partition coefficients for octanol/water.

^e^Predicted brain/blood partition coefficient.

^f^Number of hydrogen bonds donated by the molecule.

^g^Number of hydrogen bonds accepted by the molecule.

^h^Number of violations of Lipinski’s “Rule of Five”.

By analyzing the characteristics of the obtained molecules, such as: molecular weight (MW), predicted skin permeability (QP logKp), partition coefficients for octanol/water (Clog O/W), predicted brain/blood partition coefficient (Log B/B), estimated amount of hydrogen bonds‐donor (HBD) and estimated amount of hydrogen bonds‐acceptor (HBA), Lipinski′s rule of five (R5) and Jorgensen′s rule of three (R3), it was observed that all the molecules investigated here did not present any violation, except for the pivotal compound (pyriproxyfen), being in agreement with the Lipinski and Jorgensen rules [71–73], which is in line with studies carried out by Gaddaguti et al. [74]. Compounds that exhibit minimal or no deviations from these established rules possess a higher probability of successful oral administration and favorable systemic bioavailability. The individual evaluation of each characteristic of the molecules under test comprises a detailed absorption, distribution, metabolism and excretion (ADME) analysis, allowing a better understanding of the compounds. This is essential to optimize its use in formulations, in addition to identifying possible limitations and risks associated with its application. All evaluated molecules showed compliance with the reference parameters (normal range) described in Table 1, indicating that they are promising based on the aspects analyzed so far.

The toxicological aspects of the molecules that showed signs of toxicity are described in Table 3. Early identification of signs of toxicity in molecules is essential in the development of novel molecules, such as insecticides, as it allows the elimination of substances with high risks to the environment, animal, and human health, optimizing resources and research time. This assessment contributes to the development of more effective and safer molecules, ensuring greater compliance with regulatory requirements and promoting safety in the end use of the product [75].

**Table 3:** Predicted toxicological properties of molecules.

Molecules	Toxicity prediction alert (Lhasa prediction)	Toxicity alert
1YL	Skin sensitization	Plausible
BT7	No alerts	—
I40	Hepatotocity	Plausible
	Carcinogenicity: thyroid toxicity	Plausible
JHII	Skin sensitization	Plausible
	Irritation: of the eye and skin	Plausible
LMQC200	Hepatotocity	Plausible
LMQC145	No alerts	—
LMQC74‐39	No alerts	—
LMQC60	Hepatotocity	Plausible
	Carcinogenicity	Equivocal
LMQC53	No alerts	—
LMQC284	Nephrotoxicity	Equivocal
	Peroxisome proliferation	Plausible
LMQC38	Hepatotocity	Plausible
	Nephrotoxicity	Equivocal
	Methemoglobinemia	Plausible
LMQC281	Hepatotocity	Plausible
LMQC3	No alerts	—
LMQC112	Hepatotocity	Plausible
	Nephrotoxicity	Equivocal
LMQC23	No alerts	—
LMQC196	Skin sensitization	Plausible
LMQC208	No alerts	—
LMQC20	Hepatotocity	Plausible
LMQC79‐39	No alerts	—
LMQC115	No alerts	—
LMQC250	No alerts	—
LMQC184	Hepatotocity	Equivocal
LMQC46	No Alerts	—
LMQC261	Chromosome damage	Equivocal
	Methemoglobinemia	Plausible
	Carcinogenicity	Plausible
	Hepatotocity	Plausible
LMQC215	Hepatotocity	Plausible
	Nephrotoxicity	Equivocal
LMQC209	No alerts	—
LMQC229	Hepatotocity	Plausible
	Carcinogenicity	Equivocal
	Nephrotoxicity	Equivocal
LMQC244	No alerts	—
LMQC279	Hepatotocity	Plausible
LMQC264	No alerts	—
LMQC122	Peroxisome proliferation	Plausible
LMQC104	No alerts	—
LMQC255	Nephrotoxicity	Equivocal
LMQC97	Peroxisome proliferation	Plausible
LMQC8	Hepatotocity	Equivocal
LMQC271	No alerts	—
LMQC296	Skin sensitisation	Plausible
LMQC251	Hepatotocity	Plausible
	Nephrotoxicity	Plausible
	Peroxisome proliferation	Equivocal
LMQC19	Skin sensitisation	Plausible

The investigated molecules, along with the reference compounds used as controls, were systematically evaluated for potential toxicological liabilities using predictive in silico approaches. This assessment considered both the presence of structural alerts commonly associated with adverse effects and the biological plausibility of these predictions, with particular attention to the potential target organs affected in humans, nonhuman primates (monkeys), and rodents (rats). Such a cross‐species perspective is essential in early‐stage compound screening, as it provides preliminary insight into translational safety risks and supports the rational prioritization of candidates with more favorable toxicological profiles.

The analysis focused on clinically relevant endpoints frequently associated with attrition in drug and bioactive compound development, including hepatotoxicity, nephrotoxicity, skin sensitization, and carcinogenicity. These endpoints were interpreted in the context of molecular structure, predicted exposure levels, and the likelihood of systemic distribution, recognizing that liver and kidneys play central roles in xenobiotic metabolism and elimination.

Pyriproxyfen did not exhibit any significant toxicological warning signals in the predictive models applied. This favorable profile may be attributed, at least in part, to the relatively low concentration required to achieve effective interaction at the biological target site, thereby potentially limiting systemic exposure and reducing the probability of off‐target effects. The absence of structural fragments associated with major toxicity alerts further supports its safety profile within the parameters of this computational evaluation.

In contrast, several of the tested compounds (LMQC: 200, 60, 284, 38, 81, 112, 261, 215, 229, 279, 122, 97, 296, 251, 19, 196, and 20) presented plausible toxicity alerts. Most of these warnings were related to predicted hepatotoxicity and nephrotoxicity, indicating potential risks to hepatic and renal function. Additional alerts included skin sensitization, suggesting possible dermal hypersensitivity reactions, and carcinogenicity, which represents a critical concern in long‐term exposure scenarios and regulatory evaluation.

Collectively, these findings underscore the importance of integrating early toxicological screening into the compound selection workflow. Although some candidates may demonstrate promising biological activity, the presence of predicted toxicological liabilities highlights the need for structural refinement and optimization strategies aimed at mitigating safety risks prior to experimental validation.

Regarding the warning of predicted toxicity of some tested substances, the hepatotoxicity warning is linked to the ability of certain chemical substances to cause damage to the liver, which can lead to serious health problems. Early detection and assessment of hepatotoxicity are essential to guarantee the safety and efficacy of molecules and to ensure that these compounds do not become toxic compounds during pharmacokinetics [76, 77]. The nephrotoxicity warning refers to the identification of molecules likely to have adverse effects on the kidneys, which may result in significant kidney damage. This assessment is crucial in understanding the risks associated with the use of substances without prior careful analysis, reinforcing the necessity of toxicological screening in drug development [78, 79]. Carcinogenicity alerts are a fundamental tool in the evaluation of potential carcinogens. Therefore, this is an analysis of absolute importance in the assessment of cancer risk, contributing to the safety in the use of chemical compounds [79, 80]. Finally, skin sensitivity alert is crucial in the study and development of new products, since in the human body, the skin is the largest organ and plays an important role in the absorption and metabolism of chemical substances [81]. The skin is also the first defense barrier against external agents, making it essential to assess the risks of exposure to potentially dangerous substances, as well as to understand the risks of adverse effects of chemical compounds, as well as for the development of safety strategies against toxicity [76, 79].

Considering the risks associated with the use of compounds that present toxicity alerts, and the presence of a promising number of molecules free from these alerts, it was decided to disregard the previously mentioned samples with indications of toxicity. Thus, only molecules that did not present any type of toxicological alert were selected for continued analysis. Of the 35 initial molecules, 15 were selected for the next tests (LMQC: 145, 74‐39, 3, 53, 209, 244, 264, 104, 271, 23, 208, 79‐39, 115, 250 and 46) (Table 3).

### 3.3. Prediction of Bioactivity

The virtual screening performed via ROCS and EON identified the highest‐ranked hits for further evaluation. These candidates were then subjected to pharmacokinetic modeling, which narrowed the pool to 35 molecules with favorable ADME profiles; of these, only 15 were found to pose no potential toxicological risks. The PASSONLINE platform [82] was used to predict bioactivity, and the generated data is presented in Table 4. The reference ligands (pyriproxyfen, I40, 1YL, BT7 and JHIII) exhibited biological activity efficacy, confirming the literature findings [40–43].

**Table 4:** Prediction of the bioactivities of the molecules selected by virtual screening.

Molecules	Pa	Pi	Bioactivity
Pyriproxyfen	0.313	0.015	Insecticide
I40	0.105	0.064	Acetylcholine release stimulant
1YL	0.319	0.197	Acetylcholine neuromuscular blocking agent
JHII	0.336	0.011	Insecticide
BT7	—	—	No related data
LMQC3	0.134	0.040	Acetylcholine agonist
LMQC23	0.273	0.240	Acetylcholine neuromuscular blocking agent
LMQC46	0.096	0.015	Acetylcholine M1 receptor agonist
LMQC53	—	—	No related data
LMQC104	—	—	No related data
LMQC115	—	—	No related data
LMQC145	0.085	0.077	Acetylcholine agonist
LMQC208	0.306	0.006	Acetylcholine agonist
LMQC209	—	—	No related data
LMQC244	0.199	0.012	Acetylcholine agonist
LMQC250	—	—	No related data
LMQC264	—	—	No related data
LMQC271	0.573	0.034	Acetylcholine neuromuscular blocking agent
LMQC74‐39	0.111	0.045	Acetylcholine agonist
LMQC79‐39	—	—	No related data

From the data obtained by the tests performed in WebPass, satisfactory predictions were obtained for eight molecules (LMQC3, LMQC23, LMQC46, LMQC145, LMQC208, LMQC244, LMQC271, and LMQC747‐39) indicating activities linked to acetylcholine. The activity as a neuromuscular blocking agent of acetylcholine obtained in two molecules (LMQC271 and LMQC23) resembles that achieved by the standard PDB ligand compound 4M0E (1YL), highlighting LMQC271 with Pa > 0.5, being superior to other known bioactive molecules, when *P* *a* > *P* *i*. Predicting biological activity is a necessary step in the process of developing new insecticides, as it allows for the more efficient and cost‐effective identification of compounds with potential action on specific molecular targets of insects. This rational approach reduces the need for exhaustive in vitro and in vivo assays in the early stages, speeds up the selection of promising candidates, and contributes to the creation of more selective molecules with less effect on nontarget organisms and the environment. Furthermore, predictive modeling of biological activity increases the effectiveness of formulations and favors the development of innovative, safe, and sustainable insecticides [83, 84].

### 3.4. Oral Toxicity Risk Assessment

Oral toxicity prediction is an essential step in the development of new insecticides, as it allows for the early assessment of the risks that a compound may pose to human and animal health. This approach contributes significantly to the screening of safe molecules, reducing the need for testing in animal models in the early stages and optimizing the development process. Furthermore, identifying possible oral adverse effects is essential to avoid the formulation of products that may pose risks in cases of accidental exposure or environmental contamination [85–87].

Currently, pyriproxyfen is used to control vectors in the agricultural sector, given that the targets demonstrate low resistance to its effect [88]. The lethal dose values for 50% of the tested guinea pigs (*L* *D*_50_) are presented in (mg/kg) following the pesticide classification categories established by WHO [89]. These categories are based on oral *L* *D*_50_ data in rats [38, 90, 91]. The severity of the consequences is determined by the categorization of the pesticides and the exposure dose. The compounds used in this phase had no violation of Lipinski′s rule (R5), as shown in Table 2.

The predicted oral toxicity data for the candidate molecules are summarized in Table 4. For this specific stage of analysis, eight compounds were prioritized based on their superior performance and high rankings in the preceding evaluation phases. The molecules were classified according to oral toxicity, the control compound, pyriproxyfen and the ligands I40, 1YL, JHII, and BT7 presented the respective dosages: 2.000 mg/kg, 400 mg/kg, 8.000 mg/kg, 34 mg/kg, and 500 mg/kg. The tested compounds presented the following dosages: LMQC3 (1.600 mg/kg), LMQC23 (2.400 mg/kg), LMQ46 (465 mg/kg), LMQC145 (1.000 mg/kg), LMQC208 (1.000 mg/kg), LMQC244 (1.000 mg/kg), LMQC271 (200 mg/kg), and LMQC747‐39 (807 mg/kg).

Most molecules were classified as Class IV oral toxicity (300 < *L* *D*_50_ ≤ 2000), indicating moderate toxicity. The compound LMQC23 was classified as Class V (2000 < *L* *D*_50_ ≤ 5000), being the least toxic among those evaluated. On the other hand, the compound LMQC271 was classified in Class III (50 < *L* *D*_50_ ≤ 300), representing the greatest toxic potential via the oral route among all the molecules tested (see Table 5).

**Table 5:** Results of oral toxicity prediction for entry compound.

Molecule	*L* *D*_50_ (mg/kg)	Expected toxicity class
Pyriproxyfen	2000 mg/kg	IV
I40	400 mg/kg	IV
1YL	8000 mg/kg	VI
JHII	34 mg/kg	II
BT7	500 mg/kg	IV
LMQC3	1600 mg/kg	IV
LMQC23	2400 mg/kg	V
LMQC46	465 mg/kg	IV
LMQC145	1000 mg/kg	IV
LMQC208	1000 mg/kg	IV
LMQC244	1000 mg/kg	IV

*Note:* Class I: fatal by ingestion (*L* *D*_50_ ≤ 5). Class II: fatal by ingestion (5 < *L* *D*_50_ ≤ 50). Class III: toxic by ingestion (50 < *L* *D*_50_ ≤ 300). Class IV: harmful by ingestion (300 < *L* *D*_50_ ≤ 2000). Class V: can be harmful by ingestion (2000 < *L* *D*_50_ ≤ 5000). Class VI: nontoxic (*L* *D*_50_ > 5000) [39].

Since the risk of ingesting insecticides is present in both agricultural and domestic use, it would be interesting to use substances with a lower risk of oral toxicity, making them less harmful or more likely to be reversed without permanent damage in cases of accidental ingestion. Thinking about a less risky insecticide to use near mammals/humans, we used this as a parameter to exclude compounds that present a greater risk, that is, lower *L* *D*_50_ values. The best values were selected to proceed to the next step (LMQC3 and LMQC23).

### 3.5. Comparison of AChE Active Sites

A comparison of the AChE active sites revealed a conserved catalytic framework among the three species. However, there are differences in residue numbering and sequence composition. The catalytic triad is represented by Ser238, Glu367, and His480 in *D. melanogaster*. In wild‐type *A. gambiae*, Ser360, Glu359, and His600 represent it, whereas in human AChE it is represented by Ser203, Glu334, and His447 (see Table S2). The three enzymes also possess an aromatic‐rich catalytic gorge that contributes to substrate and ligand recognition. Tryptophan represented the conserved anionic site with Trp83 in *D. melanogaster*, Trp245 in *A. gambiae*, and Trp86 in human AChE. Similarly, aromatic residues at the peripheral region of the gorge are broadly conserved at the functional level. However, their sequence numbering and local environments differ among species. In particular, the *A. gambiae* structure, used in the present study (PDB 6ARY), represents an insecticide‐resistant G280S AChE variant. In this variant, the glycine residue of the oxyanion‐hole region is replaced by serine [92, 93]. This substitution may alter the steric environment surrounding the catalytic site. The comparison offers a structural explanation for why ligand enzyme interactions may occur across insect and human AChE without resulting in identical binding modes. The present docking analysis identified distinct contact patterns for LMQC3 and LMQC23 across the three proteins as described in the following section.

### 3.6. Molecular Docking Study

Molecular docking studies play an important role in the creation of new insecticides, as they allow the simulation of the interaction between candidate compounds and biological targets present in insects, such as enzymatic proteins or specific receptors. This computational process provides detailed insight into how a compound might bind to the target′s active site, helping to predict its affinity and selectivity. To validate the methodology used in molecular docking, the cocrystal ligands were subjected to the docking process until the ideal spatial conformation was obtained. The results were then compared with the crystal structures of AChE (PDB IDs 6XYU, 4M0E, and 6ARY) and JHIII (PDB ID 5V13).

To validate the molecular docking protocol, we performed redocking of the native ligands (I40, 1YL, BT7, and JHII). The accuracy of these methods was confirmed by calculating the RMSD, yielding values of 0.29, 0.37, 1.81, and 1.33 Å, respectively. According to Gowthaman et al. [94] and Hevener et al. [95], the predicted binding method through docking suggests that if the RMSD is less than 2.0 Å with respect to the crystallographic position of a given ligand, the validation is seen as satisfactory. The results are presented in Figure 4.

**Figure 4:**
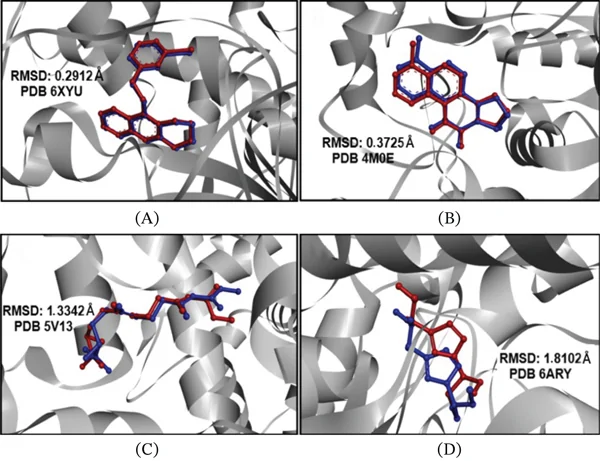
Superimpositions of crystallographic ligand poses (in blue) with the predicted poses (in red): (A) I40, (B) 1YL, (C) JHIII, and (D) BT7.

Molecular docking identified specific conformations for several ligands within their respective active sites. For I40 (PDB 6XYU), the ligand localized within an *α*‐helix defined by Tyr334, Tyr338, and Ser202, while interacting with Trp436 and Trp81 in the surrounding region. Stability was maintained via hydrogen bonding with Ser202 and Trp81, alongside hydrophobic contacts with Trp81, Trp436, Tyr338, and Tyr334. Similarly, 1YL (PDB 4M0E) occupied a site between residues Tyr329, Phe330, and Trp278, further stabilized by hydrophobic interactions with Tyr333 and Tyr121. Hydrogen bonds were notably formed with Phe287, Val286, and Tyr329. Finally, for BT7 (PDB 6ARY), the docking pose spanned an *α*‐helix and *β*‐sheet framework (including Glu197 and Ser198), supported by hydrogen bonds with Ser121, Ser198, and His438, and hydrophobic interactions with Tyr120, Phe287, Phe328, and Tyr331.Between this receptor and the molecules tested, there was a halogen interaction with the residue: Glu197. Finally, interactions with the JHIII site (PDB 5V13) were located in the *α*‐helix between the amino acid residues: Val51, Val68, and Tyr33 and around the helix the amino acid residues: Tyr129, Trp53, Phe144, and Pro55. Additionally, hydrophobic interactions with residues Trp53, Tyr129, Val51, Pro55, Val68, Tyr33, and Phe144 were observed. There was a hydrogen bond in common with residue Tyr129.

When evaluating whether the interactions of the test molecules presented greater binding affinity than the specific ligands (I40, 1YL, and BT7) of different organisms (*D. melanogaster*, *A. gambiae*, *Homo sapiens*, and *A. aegypti*), it was observed that the two compounds subjected to docking presented affinity values equal to or greater than those of the controls used (pyriproxyfen and specific ligands). To visualize the binding affinity values (*Δ* *G*) of the structures in the active sites of the respective receptors, a graph was created in heat map format (Figure 5).

**Figure 5:**
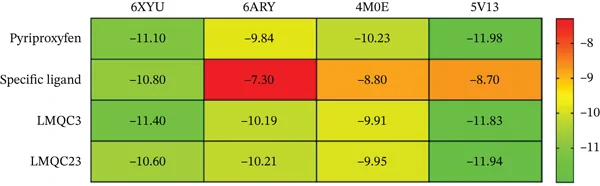
Heat map graph shows binding affinity (*Δ* *G*) values obtained via DockThor.

The molecular docking of LMQC3 and LMQC23 displayed favorable predicted interactions with AChE from *D. melanogaster* (−11.4 and −10.6 kcal/mol, respectively) and *A. gambiae* (−10.19 and −10.21 kcal/mol). The two compounds outperformed the corresponding control ligands I40 (−10.8 kcal/mol) and pyriproxyfen (−11.1 kcal/mol) (see Table 6 and Figure S1). When analyzing the docking performed based on PDB ID: 6ARY (organism: *A. gambiae*), it is observed that the compounds LMQC23 (−10.21 kcal/mol) and LMQC3 (−10.19 kcal/mol) presented higher binding affinities than the controls BT7 (−7.3 kcal/mol) and pyriproxyfen (−9.84 kcal/mol), indicating a promising potential of these compounds against the target protein of this organism (see Figure S2). It can be observed in the docking performed based on PDB ID: 4M0E (organism: *H. sapiens*), that the compounds LMQC23 (−9.95 kcal/mol) and LMQC3 (−9.91 kcal/mol) presented binding affinities higher than that of the control 1YL (−8.8 kcal/mol). However, both tested compounds exhibited slightly lower values than the control pyriproxyfen (−10.23 kcal/mol), indicating a good interaction with the human target, although with lower affinity compared with the reference compound (see Figure S3). Finally, in the docking performed based on PDB ID: 5V13 (organism: *A. aegypti*), compounds LMQC23 (−11.94 kcal/mol) and LMQC3 (−11.83 kcal/mol) showed binding affinities significantly higher than that of the control JHIII (−8.7 kcal/mol). In relation to the pyriproxyfen control (−11.98 kcal/mol), both compounds presented slightly lower values, although quite close, indicating a strong potential for interaction with the target receptor (see Figure S4). These results are within the range observed for other pyriproxyfen‐derived candidates [64] and therefore reproduce the general observation from previous studies that pyriproxyfen‐inspired compounds can occupy the AChE active site with favorable predicted binding scores.

**Table 6:** Interactions between receptors, their respective controls, and promising molecules.

	Ligand	Bindin affinity	H‐bonds	Hydrophobic interactions	Others
6XYU	I40	−10.804	SER202	TRP81, PHE335, TYR334, TYR338, TRP436	—
	LMQC3	−11.427	THR118, HIS444	TRP81, TYR334, TYR69	—
	LMQC23	−10.697	TRP436, GLU449	TRP81, TYR334, ILE80, LEU443	—

6ARY	BT7	−7.351	SER121, SER198, HIS438,	TYR120, PHE287, PHE328, TYR331	GLU197 (halogen)
	LMQC3	−10.804	GLY117, TYR120	TRP83, TYR331, TYR120, TYR327, ILE69	—
	LMQC23	−10.697	TYR120, SER198, GLU197	TYR120, SER121, TYR327, VAL70, TRP83, HIS438, ILE69	ASP71 (electrostatic)

4M0E	1YL	−8.853	PHE287, TYR329, VAL286	TRP278, TYR333, TYR121, PHE289, TYR329, PHE330	—
	LMQC3	−9.957	—	TRP278, TYR333	—
	LMQC23	−9.916	TYR69, PHE287, TYR121	TRP278, TYR333	—

5V13	JHIII	−8.701	TYR129	TRP53, VAL51, PRO55, VAL68, TYR33, TYR129, PHE144	—
	LMQC3	−11.839	SER69, TRP53, TYR133, TYR64, TYR129	TYR129, PHE144, TYR33, TRP53, VAL68, LEU74, LEU37, VAL51	—
	LMQC23	−11.948	VAL65, TYR129, SER69	TRP53, TYR33, TYR64, VAL68, TYR129, PHE144, VAL51, ALA281	

When comparing the amino acid residues present in the interaction with the test molecules and the specific control for each PDB, the repetition of these same amino acids can be seen. Table 6, Figures 6, 7, 8, and 9 and Figures S5S6, S7, and S8 show the interactions and types present for each PDB and with each molecule (test or control).

**Figure 6:**
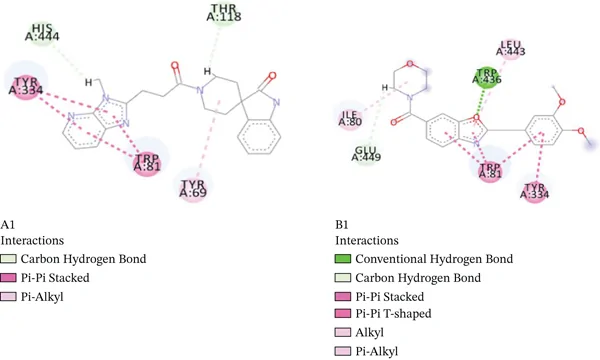
Interactions of the active site of insect AChE of *Drosophila melanogaster* with (A) LMQC3 and (B) LMQC23.

**Figure 7:**
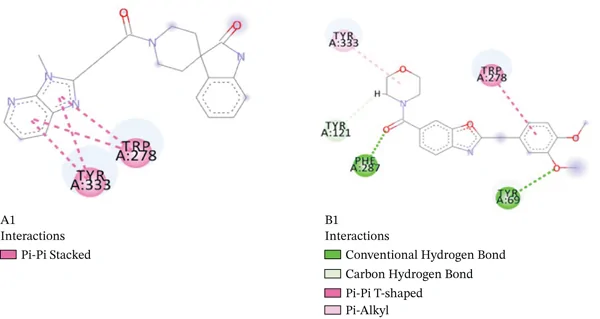
Interactions of the human AChE active site of *Homo sapiens* with (A) LMQC3 and (B) LMQC23.

**Figure 8:**
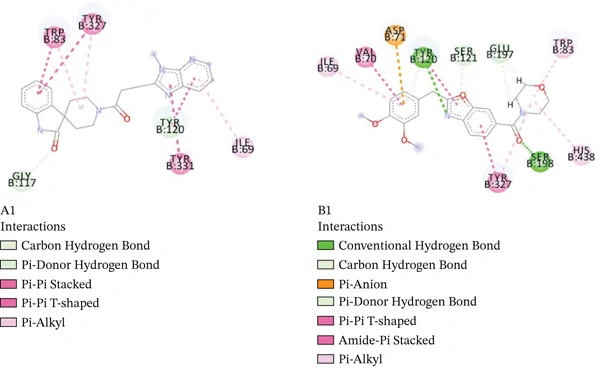
Interactions of the active site of insect AChE of *Anopheles gambiae* with (A) mLMQC3 and (B) LMQC23.

**Figure 9:**
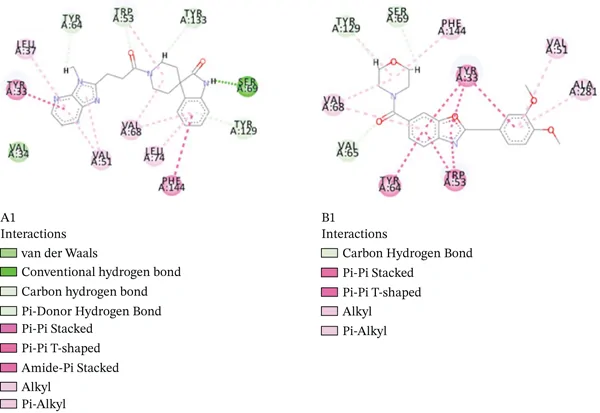
Interactions of the active site of the juvenile hormone of *Aedes aegypti* with molecules (A) LMQC3 and (B) LMQC23.

The interaction patterns further support the docking scores. In *D. melanogaster* AChE, LMQC3, and LMQC23 showed interactions with Trp81 and Tyr334, with LMQC23 additionally interacting with Tyr436 as detailed in Table 6 and illustrated in Figure 6A,B. This observation is consistent with previous docking studies in which pyriproxyfen‐derived compounds interacted with aromatic residues within the AChE active site such as Trp83 and Tyr370 in *D. melanogaster* AChE [39]. The observed aromatic interactions across different independent studies suggests that hydrophobic and *π*‐mediated interactions within the AChE active site contribute to the stabilization of pyriproxyfen‐inspired ligands. However, this should be interpreted cautiously since differences in residue numbering, different PDB structures, docking software, scoring functions, and ligand conformations can influence the predicted interaction pattern.

Compounds exhibiting insecticidal potential often act through the permanent inhibition of AChE. This enzyme plays a vital role in neurotransmission by catalyzing the hydrolysis of the neurotransmitter acetylcholine to terminate nerve impulses. As established by various researchers, the disruption of this enzymatic process is recognized as a primary mechanism of action for substances targeting the larval stage of insects. [39, 96–100]. Analyzing the interactions within the AChE active site is essential, as it features three distinct structural characteristics that are fundamental to understanding the enzyme′s biological mechanism of action.

The first relevant feature is the anionic site, which binds to acetylcholine through electrostatic interactions. Next, we have esterase, which promotes the hydrolysis of the neurotransmitter into choline and acetic acid. Finally, the catalytic domain of AChE plays a crucial role in the breakdown of acetylcholine. AChE inhibitors act directly on these sites, interrupting the normal function of the enzyme [101–104].

In the docking with PDB 4M0E, when considering the active site of AChE that binds 1YL, the compounds LMQC3 and LMQC23 had similar interactions in the *α*‐helix between the amino acid residues Trp278 (LMQC3 and LMQC23); around the helix with the residues Phe287, Tyr333, and Tyr121 (LMQC23) and Tyr333 (LMQC3) as can be seen in Figure 7A,B and Table 6.

The *A. gambiae* results are particularly relevant in the context of insecticide resistance. LMQC3 and LMQC23 showed interactions with Tyr120, Ser198, Glu197, Tyr331, Ser121, and His438 as detailed in Figure 8A, Figure 8B, and Table 6. This observation is important because it has been shown that insecticide resistance can modify the architecture of the *A. gambiae* AChE active site. The 6ARY PDB structure used in the in this study is that of an insecticide‐resistant AChE mutant. It has been structurally characterized as a model for understanding resistance‐associated changes in the active site [43] . The results, therefore, indicate that these molecules retain favorable predicted interactions with a structurally altered *A. gambiae* AChE target and therefore warrant additional experimental investigation.

Some organophosphate compounds, for example, are known to form a covalent bond with the active site of AChE, causing its irreversible inactivation. This type of inhibition results in an excessive accumulation of acetylcholine in the synapses, causing dysregulation of nerve signals and eventual death of the insects. Molecular studies and computational modeling are essential tools to understand these interactions at the atomic level and develop new compounds that are more efficient as insecticidal agents [105, 106].

Furthermore, insecticide resistance can occur through mutations in the AChE gene, alteration of the enzyme target or increased expression of detoxifying enzymes. Therefore, integrated pest management strategies should consider rotation of different classes of insecticides and ongoing research to monitor and mitigate such resistance [61–63].

In the docking with PDB 5V13, the observed interactions during the docking of the LMQC3 and LMQC23 molecules were similar to those observed for JHIII within the active site of the JH, that is, in the *α*‐helix between the amino acid residues Val51, Val68, Tyr33 (LMQC3 and LMQC23); around the helix with Tyr129, Trp53 and Phe144 (LMQC3 and LMQC23), as shown in Figure 9A,B and Table 6.

Critical life stages in insect, including growth, metamorphosis, and reproduction, are governed by JHs and ecdysteroids. These hormones are synthesized in specialized endocrine glands, namely the corpora allata and the prothoracic glands. Because of their central role in development, the receptors for these hormones have emerged as primary targets for neurotoxic and biorational insecticides. The design of modern analogs, such as methoprene and tebufenozide, relies on classical bioassays designed to quantify the hormonal agonist activity of these compounds [107–109].

These hormones play crucial roles in different stages of insect life: JHs are responsible for maintaining the larval state, whereas ecdysteroids trigger molting and metamorphosis. By interfering with this hormonal balance, biorational insecticides aim to control pests selectively, causing less environmental impact compared with traditional pesticides [107, 109].

For example, methoprene is a JH analog that prevents the transition of larvae to the adult stage, resulting in the death of the larvae. Tebufenozide, an ecdysteroid analog, induces premature and lethal molting. Furthermore, these insecticides have been successfully used to control various agricultural pests and disease vectors, contributing to crop protection and public health [109–111].

Comprehensive data regarding the binding residues, interatomic distances, interaction types, and Gibbs free energy (*Δ* *G*) for the candidate compounds against insect/human AChE and JH are detailed in Table 6 and Figures 6, 7, 8, and 9. Analysis revealed that both the reference ligands (I40, 1YL, BT7, and JHIII) and the top‐performing compounds generally exhibited an increased density of molecular interactions. This correlation resulted in a reduction of binding free energy, signifying enhanced thermodynamic spontaneity and structural stability within the complexes.

In total, approximately 47 amino acid residues participated in interactions with the tested ligands and controls. Among them, 12 residues were identified most frequently, although distributed among the different PDBs analyzed. The most recurrent residues were: Trp81, Tyr334, Tyr120, Phe287, Trp278, Tyr333, Tyr129, Trp53, Val51, Val68, Tyr33, and Phe144. Tyrosine (Tyr) was the most prevalent amino acid, appearing in different positions and in all PDBs studied, totaling 13 distinct occurrences. These findings, together with the positive binding affinity results, suggest that these residues may play an important role in the potential insecticidal activity of the analyzed compounds.

When analyzing the interactions with amino acid residues in PDB 6XYU, it was observed that compound LMQC3 presented the lowest binding free energy (−11.4 kcal/mol), even surpassing the control ligand I40 (−10.8 kcal/mol). Among the amino acid residues involved in the interactions are Tyr334 and Trp81, which were present in each ligand analyzed (LMQC3, LMQC23, and I40), indicating that these residues are consistently reactive and possibly crucial for the stabilization of interactions in the active site (Table 6). Both residues are located in regions surrounded by hydrophobic side chains and participate in *π* − *π* stacking interactions (Figure 6), a form of noncovalent attraction between aromatic rings. This type of interaction occurs when the *π* orbitals of molecules overlap, resulting in stacking forces that are biologically relevant, especially in the structural stabilization of biomolecules such as proteins [112]. Although compound LMQC23 presented a slightly lower binding free energy (−10.6 kcal/mol) than the control, its value is still quite close, which, combined with the positive results in the toxicity analyses and its good performance against other molecular targets (other PDBs), reinforces its potential as a promising compound.

In molecular docking with PDB ID 6ARY, it was observed that the most significant interactions occurred with the amino acid residue Tyr120, present in each ligand analyzed (LMQC3, LMQC23, and control BT7), as shown in Table 6. This residue is mostly surrounded by hydrophobic side chains and participates in *π* − *π* T‐shaped interactions (Figure 8), which occur when the edge of an aromatic ring approaches orthogonally the face of another ring. These interactions are recurrent in biomolecules and play a role in the structural and functional stabilization of proteins and nucleic acids [113]. Compounds LMQC3 and LMQC23 showed binding free energy values of −10.8 kcal/mol and −10.6 kcal/mol, respectively, higher than that of the control ligand BT7 (−7.3 kcal/mol).

For molecular docking performed with PDB ID 4M0E, the tested ligands, including the control 1YL, showed similar interactions with the amino acid residues Tyr333 and Trp278, both located in predominantly hydrophobic regions (Table 7). Most of these interactions were of the *π*–alkyl type (Figure 6A,B), a type of noncovalent interaction that occurs between the *π* orbital of aromatic rings and alkyl groups. Although they do not involve strong chemical bonds, these interactions are fundamental for structural stability and significantly influence the biochemical properties of molecules. The binding energies of compounds LMQC3 and LMQC23 were practically identical (−9.9 kcal/mol), both higher than that of the control ligand 1YL (−8.8 kcal/mol). Although LMQC23 formed a greater number of interactions with amino acid residues, both compounds demonstrated good performance.

**Table 7:** Synthetic accessibility values for two structures tests via SwissADME and AMBIT‐SA.

Structures	SA SwissADME	AMBIT‐SA
Pyriproxyfen	3.30	73.96
I40	2.76	71.98
1YL	3.74	62.46
BT7	2.45	82.06
JHIII	3.78	76.15
LMQC3	3.38	63.10
LMQC23	3.17	70.46

*Note:* SA scores range from 1 (*very easy*) to 10 (*very difficult*).

In the docking performed with PDB ID 5V13, a greater similarity of interactions was observed between the ligands and controls tested, with emphasis on the amino acids Tyr129, Trp53, Val51, Val68, Tyr33, and Phe144. This PDB presented the highest number of residues in common among all the ligands evaluated, compared with the other complexes (Table 6). The predominant interactions in these residues were of the *π*‐alkyl and carbon–hydrogen bond type (Figure 9), with the majority of these amino acids inserted in regions with hydrophobic side chains. The *π*–alkyl interactions, previously described, involve the overlap between *π* orbitals and alkyl groups, contributing to bond stability. Carbon–hydrogen bonds are covalent bonds between carbon and hydrogen atoms. Although widely present in organic compounds, these bonds present a very slight polarization, due to the small difference in electronegativity between the elements, and are considered weakly polar [114]. Regarding binding energy, compound LMQC23 presented the lowest value (−11.9 kcal/mol), followed closely by LMQC3 (−11.8 kcal/mol), both higher than the JHIII control (−8.7 kcal/mol). These results show that the two compounds tested have high affinity for the active site of the JH. These findings are also consistent with earlier pyriproxyfen‐based studies [64]. This close agreement supports the feasibility of using pyriproxyfen‐derived chemical similarity as a strategy for identifying potential JH inhibitors. These results also extend previous work by identifying LMQC3 and LMQC23 (Figure 10) as candidates that simultaneously display favorable predicted interactions with JH and AChE enzymes.

**Figure 10:**
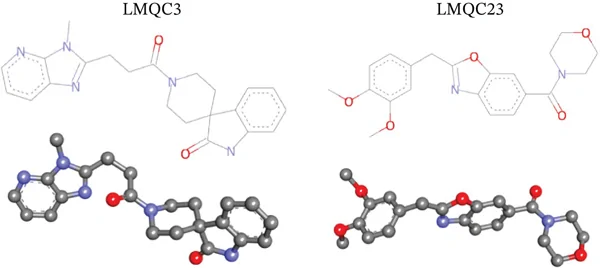
Promising molecules LMQC3 and LMQC23, selected by virtual screening.

### 3.7. SA via SwissADME and AMBIT‐SA Web Server

When analyzing the data obtained through the SwissADME platform, it was found that the LMQC3 structure presented an ease of synthesis (SA) score of 3.38, whereas LMQC23 obtained a value of 3.17 (Table 7). When compared with the control compounds (pyriproxyfen, I40, 1YL, BT7, and JHIII), whose scores varied between 2.45 and 3.78, it is observed that the values obtained for the tested structures are similar. Between the two promising compounds, LMQC23 proved to be slightly easier to synthesize. However, both compounds (LMQC3 and LMQC23) fall within the range considered synthetically accessible, according to the parameters described in the specialized literature, reinforcing their viability for development in future studies [115].

According to the results obtained through the AMBIT‐SA tool, both candidate molecules demonstrated good prospects regarding ease of synthesis, with emphasis on the LMQC23 molecule, which presented the best score. When compared with control compounds, the test molecules exhibited lower synthetic complexity values than most of the controls evaluated. These data support the predicted SA of the proposed structures. This indicates that the routes provide reasonable starting points for future experimental synthesis. However, experimental optimization will be required to establish reaction yields, reproducibility, purification efficiency, and scalability. The selection of the most promising virtual ligands to be synthesized and tested is one of the most critical aspects of computer‐coordinated drug design activities. SA is a relevant factor in this selection method, in silico prediction can be used as a prefilter when there are many molecular structures to be evaluated [115].

### 3.8. Lipophilicity and Water Solubility

With the exception of BT7 (LogP = 2.09), the control compounds (pyriproxyfen, I40, 1YL, and JHIII) exhibited highconsensus LogPo/w values in this study. The analyzed compounds are characterized as sparingly soluble in aqueous media, necessitating the use of organic solvents for effective solubilization. Specifically, pyriproxyfen is documented to be soluble in ethanol, DMSO, and dimethylformamide (DMF) [116]. Among the promising compounds evaluated in the this study, the LMQC23 structure showed the highest LogPo/w consensus value. The aromatic rings and methoxyl (‐OCH_3_) present in this molecule dominate the hydrophobic character, allowing interactions with nonpolar environments, such as lipid membranes or hydrophobic regions of proteins.

The structure of LMQC3 contains important functional groups, such as the methyl group linked to the nitrogenous ring, aromatic rings (benzene ring and aromatic heterocycle), carbonyls and hydroxyls, including forming a hydroxylamine, in addition to amides and amines that give this molecule hydrophilic and hydrophobic properties in its portions, allowing interactions with different chemical environments, such as membranes and proteins, these characteristics reflect the LogP values. In this study, the predicted LogS (SILICOS‐IT) values were considered poorly soluble with values of −6.23 and −6.16 for LMQC3 and LMQC23, respectively, and between −2.30 to 6.88 for the controls (Table 8), suggesting that all test compounds are highly lipophilic. Collectively, these findings indicate that the majority of the compounds possess moderate to low aqueous solubility, with LogS values falling within the −4 to −6 range. Such profiles signify that these molecules require organic co‐solvents to achieve complete dissolution in water‐based media.

**Table 8:** Prediction of lipophilicity and water solubility through SwissADME.

Parameters		Pivot1	140	1YL	JHIII	BT7	LMQC3	LMQC23
Lipophilicity	iLOGP	3.45	3.50	2.43	3.98	2.02	3.01	3.54
	XLOGP	4.79	5.52	3.16	3.98	1.99	1.32	2.75
	WLOGP	4.72	4.99	3.29	3.79	3.06	1.46	2.53
	MLOGP	2.98	4.52	1.93	2.72	1.44	1.86	1.44
	SILICOS‐IT	4.07	5.89	4.35	4.37	1.95	2.55	3.65
	CONSENSUS	4.00	4.88	3.03	3.77	2.09	2.04	2.78

Water Solubility	ESOL	−4.94	−6.20	−3.91	−3.47	−2.36	−3.20	−3.94
	Ali	−5.37	−5.80	−3.74	−4.50	−2.42	−2.60	−3.96
	SILICOS‐IT	−7.10	−8.64	−5.76	−3.36	−2.13	−6.23	−6.16
	CONSENSUS	−5.80	−6.88	−4.47	−3.77	−2.30	−4.01	−4.68

### 3.9. Theoretical Synthetic Route

The LMQC3 molecule can be obtained in a single synthetic step via a carboxylic acid‐amine condensation reaction, where 3‐(3‐methyl‐3H‐imidazo [4,5‐b]pyridin‐2‐yl) propanoic acid (I) is activated with oxalyl chloride (COCl₂), catalyzed by DMF in dichloromethane (DCM) at 0°C. After complete activation, the reagent spiro [indoline‐3,4’‐piperidin]‐2‐one (II) and diisopropylethylamine (DIPEA) as base are added, and the reaction is stirred at room temperature for 6 h [117], yielding the target compound (Figure 11).

**Figure 11:**
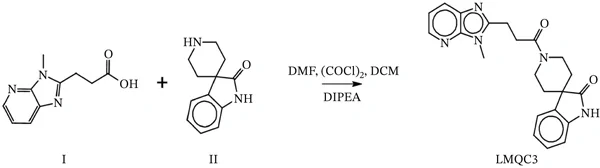
Theoretical synthetic route for the preparation of compound LMQC3.

The LMQC23 molecule can be synthesized through two consecutive steps. In the first step, 4‐amino‐3‐hydroxybenzoic acid (I) reacts with 2‐(3,4‐dimethoxyphenyl) acetic acid (II) in trimethylsilyl polyphosphate ester (PPSE) at 140°C for 2 h, followed by DCM extraction and column chromatography purification, yielding 2‐(3,4‐dimethoxybenzyl)benzo [d]oxazole‐6‐carboxylic acid (III) as a solid intermediate [118].

In the second step, the carboxylic acid intermediate undergoes an amide coupling reaction with morpholine (IV) using 1‐ethyl‐3‐(3‐dimethylaminopropyl) carbodiimide (EDC) and 1‐hydroxybenzotriazole (HOBt) as coupling agents in DCM, with triethylamine (TEA) as base. The reaction is stirred at room temperature for 24 h, followed by DCM extraction and column chromatography purification, resulting in compound LMQC23 [119] (Figure 12).

**Figure 12:**
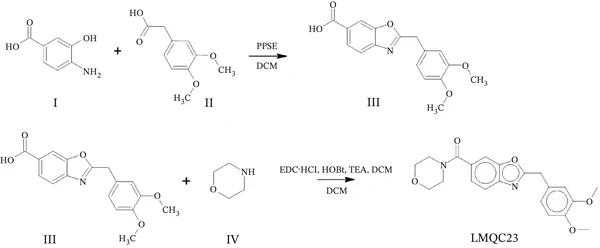
Theoretical synthetic route for the preparation of compound LMQC23.

It is important to mention that the proposed synthetic routes are prospective synthetic strategies and not experimentally validated procedures. The synthesis of LMQC3 and LMQC23 was not performed in the present study. Nevertheless, their predicted SA and the nature of the proposed transformations provide an initial assessment of their practical feasibility. LMQC3 can theoretically be obtained through a single carboxylic‐acid activation/amide‐coupling sequence, requiring COCl₂, DMF, and DIPEA under relatively conventional reaction conditions. The route therefore involves limited synthetic steps and avoids the isolation of multiple intermediates. LMQC23 requires two consecutive transformations, that is, benzoxazole formation followed by amide coupling with morpholine. Although this route is comparatively longer, both steps are based on established synthetic reactions and use reagents that are routinely used in organic synthesis. The proposed LMQC23 route also requires chromatographic purification after each step. This may increase synthetic complexity and potentially affect overall material recovery.

The SwissADME and AMBIT‐SA synthetic‐accessibility scores for LMQC3 and LMQC23 further support their predicted accessibility. These predictions, however, serve as prioritization metrics rather than experimental evidence of synthetic success. They provide reasonable starting points for future preparation and experimental validation of the two potential ligands.

## 4. Conclusions

Given the increase in insect resistance to traditional insecticides, the development of new compounds with greater selectivity for vector species and less impact on humans and the environment is becoming increasingly urgent. In this context, the market has encouraged the creation of bioinsecticides, biocides, and biopesticides, especially those derived from microorganisms or that act through hormonal modulation of the insects themselves. Molecular docking was instrumental in identifying candidates capable of competitively binding to the active sites of both AChE and the JH receptor. The study yielded promising results, highlighting structures that mimic the efficacy of pyriproxyfen while maintaining favorable pharmacokinetic and toxicological profiles—requirements that are vital for the design of potent, environmentally sustainable insecticides. Compounds LMQC3 and LMQC23 emerged as the most promising candidates in our molecular docking analysis, demonstrating robust interactions with both AChE and the JH receptor. The binding modes for these molecules closely mirrored those of the established reference standards (I40, 1YL, BT7, JHIII, and pyriproxyfen). Moreover, both leads exhibited binding free energies that were either superior or comparable with those of the control group. Given their consistently high performance throughout the in silico pipeline, these molecules represent promising insecticidal agents; consequently, our group is now focused on developing specific formulations for subsequent in vivo validation.

## Author Contributions

Conceptualization: Njogu M. Kimani, José M. Espejo‐Román, and Cleydson. B.R. Santos. Methodology: Edla L.V.S. dos Santos, Ryan da S. Ramos, Luciane B. da Silva, Ruan S. Bastos, Aderaldo V. da Silva, Sylviah S. Mwanza, Njogu M. Kimani, and José M. Espejo‐Román. Software: Ryan da S. Ramos, Joaquín M.C. Rosa, and Raimundo N.P. Souto. Data curation: Edla L.V.S. dos Santos, Rai C. Silva, and Cleydson. B.R. Santos. Investigation: Rai C. Silva, Luciane B. da Silva, Ruan S. Bastos, Joaquín M.C. Rosa, and Raimundo N.P. Souto. Validation: Edla L.V.S. dos Santos, Rai C. Silva, Lúcio R. Lima, Aderaldo V. da Silva, and Joaquín M.C. Rosa. Formal analysis: Edla L.V.S. dos Santos, Ryan da S. Ramos, Ruan S. Bastos, and Joaquín M.C. Rosa. Supervision: Njogu M. Kimani, José M. Espejo‐Román, Raimundo N.P. Souto, and Cleydson. B.R. Santos. Visualization: Lúcio R. Lima. Project administration: Cleydson. B.R. Santos. Resources: Raimundo N.P. Souto. Writing—original draft: Edla L.V.S. dos Santos, Ryan da S. Ramos, Lúcio R. Lima, Ruan S. Bastos, Aderaldo V. da Silva, and Cleydson. B.R. Santos. Writing—review and editing: Ryan da S. Ramos, Rai C. Silva, Luciane B. da Silva, Sylviah S. Mwanza, Njogu M. Kimani, José M. Espejo‐Román, Raimundo N.P. Souto, and Cleydson. B.R. Santos.

## Funding

No funding was received for this manuscript.

## Conflicts of Interest

The authors declare no conflicts of interest.

## Supporting information


**Supporting Information** Additional supporting information can be found online in the Supporting Information section. Figure S1: Results of the binding affinity of the compounds with I40 (PDB ID: 6XYU). Figure S2: Results of the binding affinity of compounds with BT7 (PDB ID: 6ARY). Figure S3: Results of the binding affinity of the compounds with 1YL (PDB ID: 4M0E). Figure S4: Results of the binding affinity of the compounds with JHIII (PDB ID: 5V13). Figure S5: Interactions of the active site of insect acetylcholinesterase (*Drosophila melanogaster*) with (A) the molecules LMQC3 3D and (B) molecules LMQC23 3D. Figure S6: Interactions of the human acetylcholinesterase active site of *Homo sapiens* with (A) LMQC3 molecules 3D and (B) molecules LMQC23 3D. Figure S7: Interactions of the active site of insect acetylcholinesterase (*Anopheles gambiae*) with (A) LMQC3 molecules 3D and (B) molecules LMQC23 3D. Figure S8: Interactions of the active site of the juvenile hormone of *Aedes aegypti* with (A) the molecules LMQC3 3D and (B) molecules LMQC23 3D. Table S1: Structural, physical, chemical and biological properties of pyriproxyfen. Table S2: Comparative analysis of the principal active‐site residues of AChE from *Drosophila melanogaster*, *Anopheles gambiae*, and *Homo sapiens*.

## Data Availability

The data that supports the findings of this study are available in the Supporting Information of this article.
