# Supplementary material for: Scaffold‐Hunting From Pyriproxyfen: A Biochemoinformatic Route to Novel Insecticidal Leads
Source: Biomed Res Int. 2026 Oct 21;2026:6490830. doi: 10.1155/bmri/6490830 (PMC13687793; doi:10.1155/bmri/6490830)
Supplement: Supplementary file 1 [file BMRI-2026-6490830-s001.docx]

**Supplementary Materials**

**Figure S1.** Results of the binding affinity of the compounds with I40 (PDB ID: 6XYU).

**Figure S2.** Results of the binding affinity of compounds with BT7 (PDB ID: 6ARY).

**Figure S3.** Results of the binding affinity of the compounds with 1YL (PDB ID: 4M0E).

**Figure S4.** Results of the binding affinity of the compounds with JHIII (PDB ID: 5V13).

**Figure S5**. Interactions of the active site of insect acetylcholinesterase (*Drosophila melanogaster*) with the molecules LMQC3 3D (A) and molecules LMQC23 3D (B).

**Figure S6.** Interactions of the human acetylcholinesterase active site of *Homo sapiens* with LMQC3 molecules 3D (A) and molecules LMQC23 3D (B).

**Figure S7.** Interactions of the active site of insect acetylcholinesterase (*Anopheles gambiae*) with LMQC3 molecules 3D (A) and molecules LMQC23 3D (B).

**Figure S8.** Interactions of the active site of the juvenile hormone of *Aedes aegypti* with the molecules LMQC3 3D (A) and molecules LMQC23 3D (B).

Table **S1**: Structural, Physical, chemical and Biological properties of pyriproxifen

| **Property Category** | **Parameter / Feature** | **Description / Value** |
| --- | --- | --- |
| **Structural Properties** | Chemical Name (IUPAC) | 2-[1-methyl-2-(4-phenoxyphenoxy)ethoxy]pyridine |
|  | CAS Registry Number | 95737-68-1 |
|  | Molecular Formula | C20H19NO3 |
|  | Molecular Weight | 321.37 g/mol |
|  | Chemical Class | Pyridine-based Insect Growth Regulator (Phenoxy Juvenile Hormone Mimic) |
|  | Structural Features | Consists of a pyridine moiety connected via an ether linkage with a methyl-substituted ethoxy spacer to a 4-phenoxyphenoxy group. |
| **Physical Properties**  **]** | Physical State & Appearance | Solid, white-to-pale-yellowish crystalline powder or waxy solid |
|  | Melting Point | 45.0 – 47.0 °C |
|  | Boiling Point / Thermal Stability | Decomposes before boiling; stable under ambient storage conditions |
|  | Water Solubility | Low solubility in water (~0.367 mg/L at 20–25 °C) |
|  | Organic Solvent Solubility | Highly soluble in organic solvents (e.g., acetone, acetonitrile, hexane, methanol, xylene) |
|  | Vapor Pressure | Low volatility (~1.42 × 10⁻5 Pa at 25 °C / ~1.0 × 10⁻7 mmHg) |
|  | Partition Coefficient (Log Kow) | High lipophilicity (Log Kow = 5.37 at 25 °C) |
| **Chemical Properties** | Hydrolytic Stability | Highly stable to hydrolysis across environmental pH levels (pH 4, 7, and 9) |
|  | Aqueous Photolysis | Susceptible to rapid photolytic degradation in surface water exposed to sunlight (DT50 = 16 to 21 days depending on solar light intensity) |
|  | Soil Adsorption / Mobility (Koc) | High adsorption affinity to soil organic carbon (Koc = 18,300 – 31,000 mL/g); classified as immobile to low-mobility, posing minimal groundwater leaching risk |
|  | Environmental Degradation / Fate | Aerobic soil half-life (DT50) ranges from 6 to 10 days; aerobic aquatic half-life ranges from 16 to 21 days; breaks down into 4'-OH-Pyriproxyfen, PYPAC, and bound residues |
| **Biological Properties** | Mode of Action (MOA) | Juvenile Hormone Mimic / Analogue (IRAC Group 7C); disrupts insect metamorphosis, inhibits adult emergence, suppresses embryogenesis, and induces adult sterility |
|  | Target Pest Spectrum | Effective against Diptera (mosquitoes, midges), Hemiptera (whiteflies, scale insects, aphids), Siphonaptera (fleas), and Thysanoptera (thrips) |
|  | Mammalian Toxicity | Low acute toxicity via oral (LD50 > 5000 mg/kg in rats), dermal (LD50 > 2000 mg/kg), and inhalation routes; non-carcinogenic and non-mutagenic |
|  | Ecotoxicity - Aquatic Organisms | Highly toxic to non-target aquatic invertebrates (e.g., Daphnia magna EC50 ~ 0.40 mg/L) and fish (LC50 ~ 0.45 mg/L in rainbow trout) upon acute exposure |
|  | Ecotoxicity - Terrestrial / Avian | Low toxicity to birds (e.g., Mallard duck oral LD50 > 2000 mg/kg) and low toxicity to adult honeybees (Apis mellifera) |

**Table S2.** Comparative analysis of the principal active-site residues of AChE from Drosophila melanogaster, Anopheles gambiae, and Homo sapiens

| **Functional region** | **D. melanogaster (6XYU)** | **A. gambiae (6ARY)** | **H. sapiens (4M0E)** | **Conservation status** |
| --- | --- | --- | --- | --- |
| Catalytic triad – Ser | Ser238 | Ser360* | Ser203 | Conserved catalytic function |
| Catalytic triad – Glu | Glu367 | Glu359 | Glu334 | Conserved catalytic function |
| Catalytic triad – His | His480 | His600 | His447 | Conserved catalytic function |
| Anionic/π-cationic site | Trp83 | Trp245 | Trp86 | Conserved aromatic function |
| PAS | Tyr71, Tyr370, Tyr374 | Tyr291, Tyr489, Tyr493 | Tyr72, Tyr337, Tyr341 | Conserved aromatic character with species-specific substitutions |
| Aromatic gorge | Phe330 | Phe490 | Phe338 | Conserved aromatic character |
| Oxyanion hole | Gly150, Gly151, Ala239 | Gly278, Gly279, Ser280* | Gly121, Gly122, Ala204 | Partially conserved; G280S distinguishes 6ARY |

***** 6ARY represents the insecticide-resistant G280S A. gambiae AChE variant; Ser360 is the catalytic serine, whereas Ser280 is the resistance-associated substitution in the oxyanion-hole region.
